# The effects of statins in patients with advanced-stage cancers - a systematic review and meta-analysis

**DOI:** 10.3389/fonc.2023.1234713

**Published:** 2023-08-18

**Authors:** Qiang Zhou, Zhihua Jiao, Yuxi Liu, Peter N. Devreotes, Zhenyu Zhang

**Affiliations:** ^1^ Department of Administration, Shenzhen Center for Prehospital Care, Shenzhen, China; ^2^ Department of Cell Biology, School of Medicine, Johns Hopkins University, Baltimore, MD, United States; ^3^ Preventive Medicine, School of Public Health, China Medical University, Shenyang, Liaoning, China; ^4^ Department of Global Health, Peking University School of Public Health, Beijing, China; ^5^ Institute for Global Health and Development, Peking University, Beijing, China

**Keywords:** Advanced-stage, Cancer, Statins, Overall survival, Meta-analysis

## Abstract

**Background:**

Statin therapy has been shown to reduce mortality in a wide range of cancer types and overall stages. Still, there is uncertainty about its efficacy in increasing survival among advanced cancer patients.

**Methods:**

We conducted a meta-analysis with data from all studies that compared the hazard ratio of overall survival, cancer-specific survival, and progression-free survival in patients with advanced-stage cancer who receive statin therapy. Studies were selected from the PubMed, Embase, and Web of Science databases from their inception to December 31, 2022. Cancer types are limited to those rarely screened during the annual examination and more likely to develop into advanced stages, such as lung, pancreatic and ovarian cancers. This resulted in 27 studies eligible for meta-analysis.

**Results:**

Statin therapy was associated with a 26% decreased risk of overall survival (HR, 0.74; 95% CI, 0.67, 0.81), 26% decreased risk of cancer-specific survival (HR, 0.74; 95% CI, 0.61-0.88), and 24% decreased risk of progression-free survival (HR, 0.76; 95% CI, 0.65-0.87) for advanced-stage cancer patients. The associations were not attenuated or reinforced by study design, study regions, cancer types, or other medical care. Concomitant use of other anticancer medications did not result in confounding effects.

**Conclusions:**

Statin therapy produces significant benefits on overall survival and cancer-specific survival. Although the benefits might be lower than the approved immunotherapy medications, its cost-effectiveness could lead to dramatic health consequences. Concomitant use of statin drugs as cancer treatments is highly recommended in future clinical trials.

## Introduction

Statins, also known as 3-hydroxy-3-methyl-glutaryl-coenzyme A (HMG-CoA) reductase inhibitors, are a class of cholesterol-lowering medications that reduce the risk of cardiovascular diseases. Statin use in the US has dramatically increased since lovastatin was approved by the Food and Drug Administration (FDA) in 1987 (1). In addition to their clinical benefits for cardiovascular events, statins have been widely investigated for cancer outcomes (2–6). In 1996, an increased incidence of breast cancer in patients given pravastatin was seen in the CARE trial (2). Afterward, consolidated results from several experimental studies and large, high-quality randomized trials demonstrated that statins had beneficial effects on cancer prevention (5, 7, 8).

Statins inhibit HMG coenzyme reductase, which converts HMG coenzyme A to mevalonate, and, reduces the availability of cholesterol and isoprenoids (9). *In vitro* studies have suggested that these metabolites play a vital role in cancer cell proliferation (10–12). On the one hand, cholesterol present in membrane microdomains is reported to be a prominent mediator of the Akt signaling pathway in cancer cells, which contributes to cell survival (13, 14). On the other hand, isoprenoids, especially geranylgeranyl diphosphate (GGPP), are required for the posttranslational modification of proteins that localize to the membrane. Some of these proteins, such as Rap, Rho and certain Ras proteins, are involved in networks essential for cancer cell survival (15–17). Moreover, the inhibitory effects of statins, and in particular GGPP depletion, on tumor suppression were observed in human cell lines and mouse models (17, 18). The beneficial effects of statins on inhibiting proliferation or killing cancer cells provide a molecular basis for the potential application of statins for cancer prevention in patients.

Statins have been shown to reduce cancer-related mortality (7, 19). However, few clinical trials and observational studies have evaluated the protective effects of statins in patients with advanced-stage caner (20–25). It has been reported that statin regimens were associated with prolonged on median survival (18 months) compared with the non-statin users (9 months) in patients with advanced hepatocellular carcinoma in a randomized controlled trial on overall survival (25). However, others have reported that statins use was not associated with overall survival in patients with advanced hepatocellular carcinoma and ovarian cancer (21, 24). Because there are conflicting reports, meta-analyses that only include patients with advanced-stage cancer are needed to clarify the association between statins and mortality from advanced-stage cancer. Currently, one meta-analysis is available to assess the effects of statins on advanced cancer mortality, however, it is restricted to prostate cancer with androgen deprivation therapy and only includes retrospective studies (26). Aiming to more fully understand how statins influence mortality in patients with advanced-stage cancer, we conducted a meta-analysis by collecting data from patients with advanced cancer (higher than stage 3 or metastatic) according to the American Joint Committee on Cancer staging manual (AJCC) staging system and then investigated the overall effects of statin therapy. Cancer types are limited to those that are rarely screened during annual examination and more likely to develop into advanced stages.

## Materials and methods

### Search strategy and selection criteria

**Figure 1:**
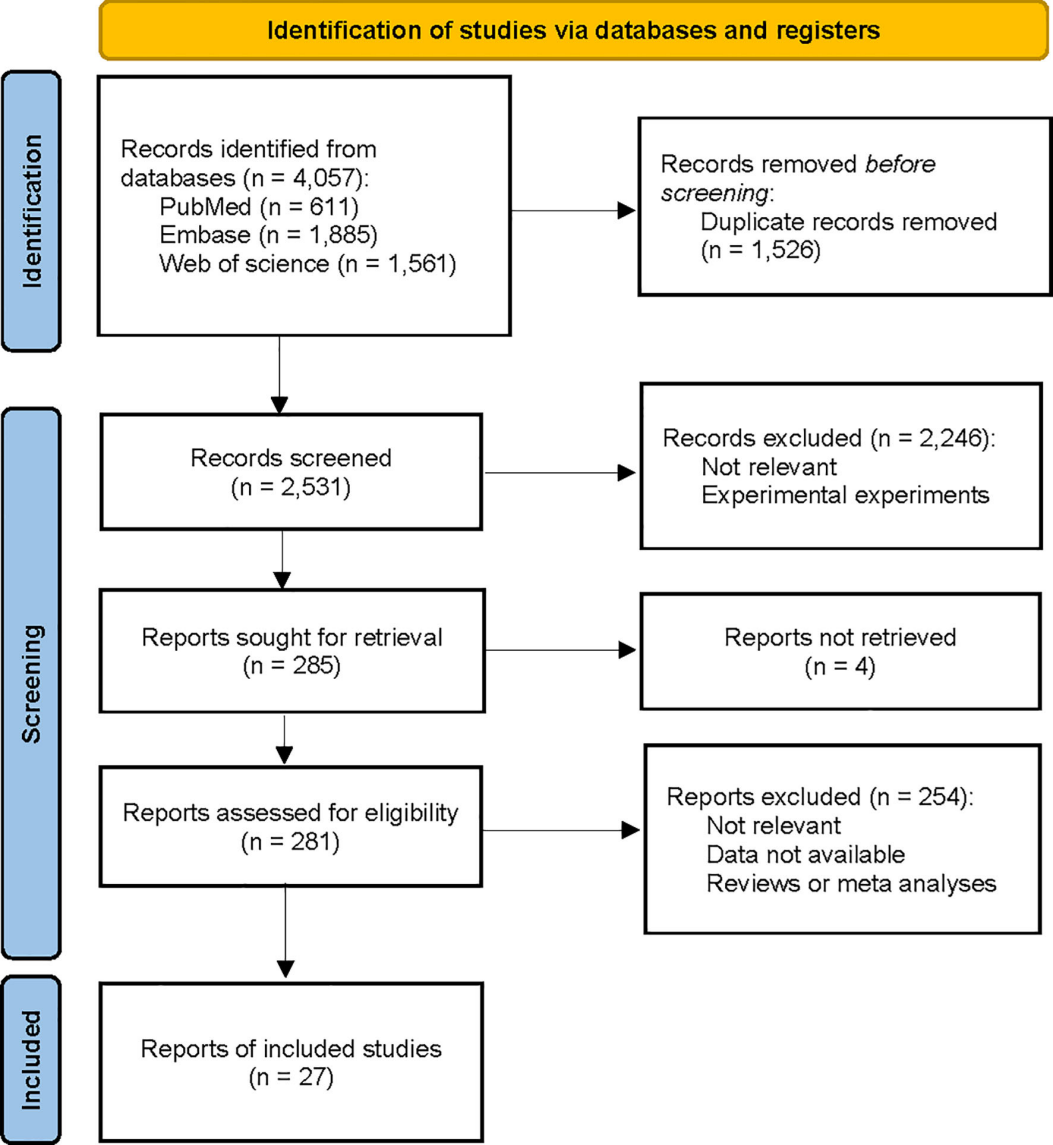
Study selection for the effect of statin on patients with advanced-stage cancer.

We conducted a comprehensive search of the PubMed, Embase, and Web of Science databases from inception to December 31, 2022 (**Figure 1**). A combination of MeSH terms and text words was used to identify published papers on the assessment of statin use and survival in advanced cancer. The search strategy is shown in **eAppendix 1**. In addition, we hand-searched the bibliographies of selected papers to identify additional relevant studies. No study design or language restriction was applied. Studies were selected based on the inclusion and exclusion criteria presented in **Textbox 1**.

Title and abstract screening were performed using Covidence. The full text of the selected studies was reviewed to determine the eligibility of inclusion. Data extraction and risk of bias analysis were performed independently by two authors (YXL and ZHJ), with any disagreements resolved by consensus.

### Data extraction and quality assessment

Data from each study were extracted using a standardized form, which included information on study characteristics (study design, source, time period, sample size), cancer (cancer type and stage), and drugs (statin type and dose). We contacted the authors of the original papers if some information was missing or unclear.

We assessed the risk of bias using the Newcastle-Ottawa scale for observational studies (27), based on the items of selection, comparability of groups, and exposure/outcome assessment. We applied criteria developed by the US Preventive Services Task Force Procedure (USPSTF12) to rate the quality of RCTs based on randomization methods, double-blind designs, and follow-up reporting (28). More detailed information on the quality assessment is available in the **Supplementary Material**.

### Data synthesis and analysis

Data abstracted included the year of publication, country, number of patients, study period, study design, cancer types, cancer stage, statin generic name, follow-up time, and primary outcomes. We conducted separate analyses for statin usage and overall survival, cancer-specific survival, and progression-free survival among patients with advanced-stage cancer. For studies combining statins with another treatment to improve overall survival, we recalculated the independent effect of statins on the survival rate. Because of the expected heterogeneity in population characteristics and study methodology, a Q statistic with a value of p <.1 or an I (2) statistic > 50% was considered to indicate significant heterogeneity between studies. If significant heterogeneity was present, a random-effects model of analysis was used; otherwise a fixed-effects model of analysis was used to combine hazard ratios to account for both between- and within-study variability.

We assessed publication bias graphically using funnel plots and statistically using Eggers’ test. We assessed heterogeneity using the *I (* 2 *)* statistic. We used subgroup analysis to determine sources of heterogeneity. Subgroup analysis included study design (RCT vs. observational studies), statin types (hydrophilic vs. lipophilic), study regions (the United States vs. European vs. Asian countries), cancer types (digestive system cancer vs. respiratory system cancer vs. reproductive system cancer), or treatment regimen (use of statins alone vs. statins combined with other medications). Statistical tests were 2-sided, and we used a significance threshold of *P* <.05. Statistical analyses were performed using the meta module of STATA MP, version 16 (Stata Corp LP, College Station, TX).

## Results

### Literature search results

We identified 3,877 relevant randomized controlled trials and prospective and retrospective cohort studies by searching three databases and reviewing relevant bibliographies. We excluded 1,346 duplicate articles and an additional 2,250 articles that did not fulfill the selection criteria. After reviewing the full text of the remaining 281 articles, 254 were excluded for several reasons, as shown in **Figure 1**. We included 27 randomized controlled trials and observational studies in the final analyses.

### Characteristics of identified trials

The included studies involved a total of 163,005 participants from more than 17 countries and consisted of 3 randomized controlled trials and 24 observational studies reported from April 2001 through December 2022. Among the studies, the median follow-up period was 41.2 months (ranging from 3.1 to 87.6 months), with a daily dose of statin ranging from 10 mg to 40 mg (**Table 1**). All three randomized controlled trials were designed with simvastatin (29–32). Among 24 observational studies, one observational study used pravastatin as the single agent (23). All the other observational studies described several statins in their research and one study evaluated the effects of each generic statin separately on survival rates (33).

**Table 1:** Characteristics of studies about the association between statins and advanced-stage cancer survival.

Author, year, study design,	Study country, study period	Number of patients (cases/control)	Cancer type	Cancer stage	Statin brand	Dose/day	Follow-up time	Pre or post diagnose
Elmore, 2008, observational	United States, 1996-2001	126 (17/109)	Ovarian cancer	III, IV	NA	NA	NA	NA
Han, 2011, RCT	Korea, 2006-2008	106 (52/54)	Lung cancer	IIIB, IV	Simvastatin	40 mg	30 m	Post
Kim, 2014, RCT	Korea, 2009-2012	244 (120/124)	Gastric cancer	IV	Simvastatin	40 mg	NA	Post
Nakai, 2013, observational	Japan, 2001-2011	250 (30/220)	Pancreatic cancer	Locally advanced or metastatic	NA	NA	9.9 m	NA
Jeon, 2015, observational	United States, 2007-2009	954 (314/640)	Pancreatic adenocarcinoma	Grade III, IV	Fluvastatin, lovastatin, pravastatin, rosuvastatin, simvastatin	NA	3.1 m	Post
Jung, 2015, observational	Korea, 1997-2013	171 (46/125)	Prostate cancer	Metastasis	NA	NA	52 m	NA
Shao, 2015, observational	Taiwan, 2001-2010	20,200 (1,988/18,212)	Hepatocellular carcinoma	III, IV	NA	NA	1.7 y	NA
Boegemann, 2016, observational	German, 2010-2015	108 (21/87)	Prostate cancer	Metastatic	NA	NA	20 m	NA
Bujanda, 2016, observational	Spain, 2009-2015	60 (20/40)	Gastric cancer	III, IV	Pravastatin	40 mg	4-6 y	Post
Chen, 2016, observational	China, 2009-2013	60 (30/30)	Ovarian cancer	III, IV	NA	10–20 mg	30.3 ± 14.9 m	NA
Lin, 2016, observational	United States, 2007-2009	5,118 (1,404/3,714)	Lung cancer	IV	NA	NA	NA	Both
Moon, 2016, observational	Korea, 2006-2014	180 (17/163)	Pancreatic cancer	III, IV	Atorvastatin, rosuvastatin, simvastatin, or pitavastatin	30 mg	NA	NA
Lam, 2017, observational	United states, 2000-2010	276 (59/217)	Lung cancer	IIIA, IIIB	NA	NA	4.7 y	NA
Lee, 2017, RCT	Korea, 2012-2015	68 (36/32)	Lung cancer	IIIB, IV	Simvastatin	40 mg	22.3 m	Post
Gordon, 2018, observational	United states, 2011-2016	598 (199/399)	Prostate cancer	Metastatic	Atorvastatin, lovastatin, pravastatin, rosuvastatin, simvastatin, unknown	30 mg	NA	NA
Hamada, 2018, observational	United states, 2000-2013	374 (139/235)	Pancreatic cancer	Metastatic	NA	NA	NA	Pre
Lorenzo, 2018, observational	Italy, 2011-2016	185 (71/114)	Prostate cancer	Metastatic	NA	NA	NA	NA
Seliger, 2018, observational	German, 1998-2013	1,093 (122/971)	Glioma	III, IV	Simvastatin, atorvastatin, cerivastatin, fluvastatin, lovastatin, pravastatin	NA	7.3 y	NA
Wu, 2019, observational	Taiwan, 2008-2014	5,749 (2,171/3,578)	Prostate cancer	T3, T4, N1, M1	Simvastatin, pitavastatin, atorvastatin, fluvastatin, lovastatin, pravastatin, rosuvastatin	NA	3.6 y	Post
Kumar, 2020, observational	United States, 2000-2015	6854 (3747/3107)	Prostate cancer	III, IV	NA	NA	5.9 y	Post
Gonzalez, 2020, observational	United states, 2010-2014	534 (128/406)	Ovarian cancer	IIIC, IV	NA	NA	NA	NA
Khan, 2021, observational	United States, 1999-2013	4556 (2088/2468)	Prostate cancer	T4, N1, M1	NA	NA	4.5 y	Post
Dighe, 2021, observational	United States, 2003-2019	141 (60/81)	Esophageal adenocarcinoma	IV	NA	NA	NA	Pre
Lopez, 2021, observational	United States, 2007-2011	110156 (1078/109078)	Prostate cancer	Advanced stages	NA	NA	5.6 y	Pre
Santoni, 2022, observational	Italy, Spain, 2016-2021	304 (93/211)	Renal cell carcinoma	Metastatic	NA	NA	35.8 m	Post
Min, 2022, observational	China, 2010-2019	4150 (219/3931)	Brain	Metastatic	NA	NA	NA	Post
Takada, 2022, observational	Japan, 2016-2019	390 (337/53)	Non−small−cell lung cancer	Advanced stages	NA	NA	457 d	NA

Advanced stages: higher than stage 3 or locally advanced, metastatic for pancreatic cancer.

The studies evaluated the effects of statin usage among patients with advanced-stage cancer. There are eight studies for patients with the advanced stage prostate cancer (6, 33–39), four for the advanced stage pancreatic cancer (40–43), five for advanced stage lung cancer (29, 31, 44–46), one for advanced hepatocellular carcinoma (47), three for the advanced stage ovarian cancer (24, 48, 49), two for advanced stage gastric cancer (23, 30), one for advanced stage esophageal adenocarcinoma (50), one for advanced stage glioma (51), one for advanced renal cell carcinoma (52), and one for cancer patients with brain metastasis (52, 53).

### Quality evaluation

Among the studies, the mean quality score evaluated by the New Castle-Ottawa Scale was 8.0 for the observational studies (**Table S1**), with 9 points for nine studies, 8 points for six studies, and 6-7 points for nine studies that had lower scores for outcome assessment. Among the clinical trials, there were two studies rated as “good” and two studies rated as “fair” on the scale by the US Preventive Services Task Force Procedure (**Table S2**). The “good” RCT studies generally used appropriate randomization methods in the study design, while the “fair” ones consisted of some limitations in study design, quality or precision.

### Primary analysis

**Figure 2:**
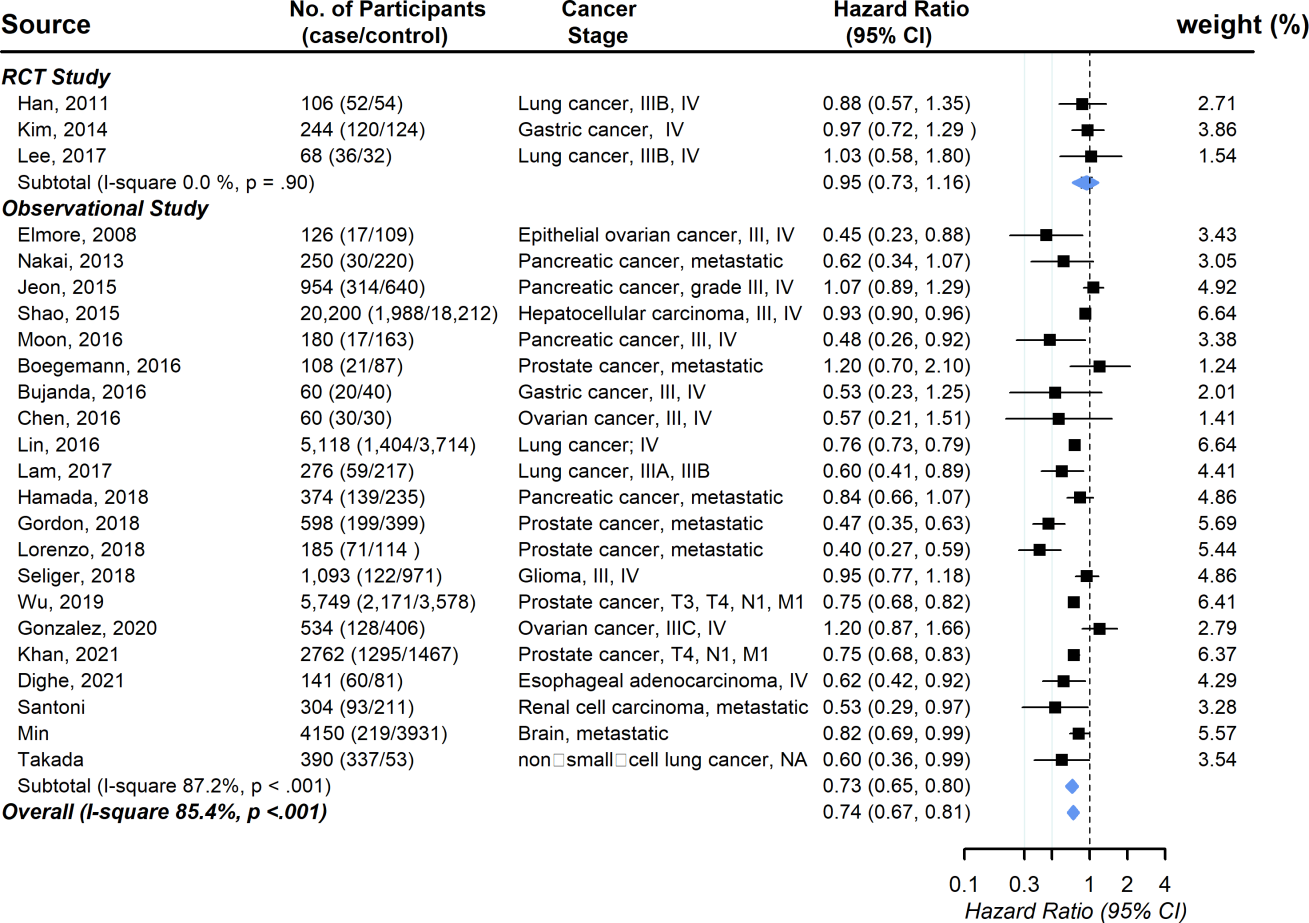
Pooled associations between statin and overall survival in patients with advanced-stage cancer.

Three RCT studies and twenty-one observational studies provided information on the association between statin usage and the overall survival rate among advanced-stage cancer patients. The pooled hazard ratio showed a significantly increased chance of overall survival (HR, 0.74; 95% CI, 0.67-0.81), with evidence of substantial between-study heterogeneity (*I* (2 *)* 85.4%, *P* <.001; **Figure 2**). Publication bias was not observed (the funnel plot was symmetric, and Egger’s test *P* = .20) (**Figure S1**). No individual study affected the overall estimate by more than 10% (**Table S3**).

**Figure 3:**
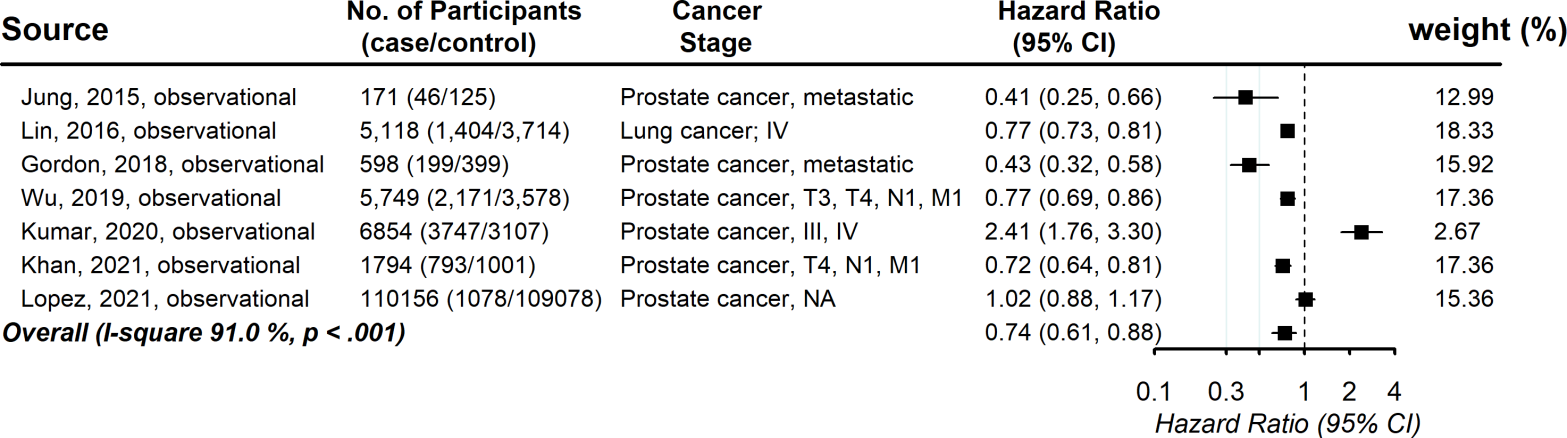
Pooled associations between statin and cancer-specific mortality in patients with advanced-stage cancer.

Seven studies provided information on the association between statin usage and the cancer-specific survival rate among advanced-stage cancer patients. The pooled hazard ratio showed a significantly increased chance of cancer-specific survival (HR, 0.74; 95% CI, 0.60-0.89), with evidence of between-study heterogeneity (*I* (2) 91.0%, *P* <.001; **Figure 3**). Visual inspection of the funnel plots revealed some asymmetry, but Egger’s tests for asymmetry were not statistically significant (*P* = .92) (**Figure S2**). As the power of Egger’s test will be low with small numbers of studies, we used trim-and-fill analysis to impute the omitted studies. The imputed estimation was consistent with the main result, with a pooled hazard ratio of 0.85 (95% CI, 0.48-1.22) (**Figure S3**). No individual study affected the overall estimate by more than 10% (**Table S4**).

**Figure 4:**
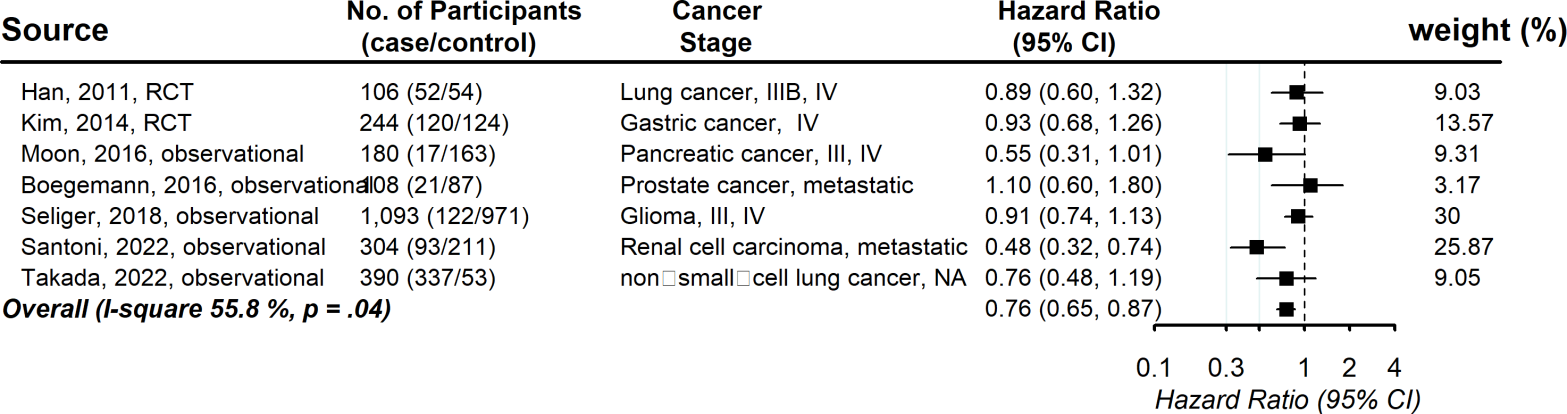
Pooled associations between statin and progression-free survival in patients with advanced-stage cancer.

Seven studies provided information on the association between statin usage and the progression-free survival rate among advanced-stage cancer patients. The pooled hazard ratio showed that the increased chance of progression-free survival was 0.76 (95% CI, 0.65, 0.87), with evidence of between-study heterogeneity (*I* (2 *)* 55.8%, *P* = .04; **Figure 4**). Publication bias was not observed (Funnel plot is symmetric and Egger’s test *P* = .59) (**Figure S4**). No individual study affected the overall estimate by more than 10% (**Table S5**).

### Subgroup analysis

We performed a number of subgroup analyses according to study design, statin types, study regions and study quality (**Table 2**). In the analysis of overall survival, there were statistically significant differences between different study designs, and the HR of the observational study was lower (0.73 vs 0.95). Because the effects of statins on the survival rate might change with cancer type, we also conducted a subgroup analysis among the digestive system, respiratory system, reproductive system and others. No statistically significant differences were demonstrated. In addition, in some of the selected studies, patients were allocated statins and other medications, including the simultaneous use of aspirin. Even though we calculated the independent effect of statins, there might be the possibility of confounding effects. As a result, we also performed subgroup analysis of the treatment regimen (use of statins alone vs statins combined with other medications). No statistically significant differences were demonstrated in any of the three survival analyses.

**Table 2:** Subgroup analyses in subset of included studies according to baseline characteristics.

Study characteristics	Overall survival		*p*	Cancer-specific survival		*p*	Progression free survival		*p*
	n	HR (95% CI)		n	HR (95% CI)		n	HR (95% CI)	
Study Design			.001			–			.22
RCT	3	0.95 (0.73, 1.16)		0	–		2	0.91 (0.69, 1.14)	
Observational	21	0.73 (0.65, 0.80)		7	0.74 (0.61, 0.88)		5	0.72 (0.51, 0.93)	
Statin types			.01			.65			–
Hydrophilic	2	0.62 (0.52, 0.71)		1	0.69 (0.57, 0.82)		0	–	
Lipophilic	5	0.86 (0.73, 0.99)		1	0.79 (0.61, 0.97)		2	0.91 (0.69, 1.14)	
Statin types			.001			.08			.22
Simvastatin	5	0.94 (0.81, 1.07)		1	0.91 (0.70, 1.13)		2	0.91 (0.69, 1.14)	
Pravastatin	2	0.56 (0.39, 0.73)		1	0.69 (0.45, 0.94)		0	–	
Atorvastatin	1	0.77 (0.67, 0.87)		1	0.74 (0.62, 0.87)		0	–	
Fluvastatin	1	0.84 (0.65, 1.04)		1	0.95 (0.68, 1.22)		0	–	
Lovastatin	1	0.97 (0.73, 1.21)		1	0.88 (0.60, 1.17)		0	–	
Rosuvastatin	1	0.64 (0.53, 0.75)		1	0.69 (0.55, 0.84)		0	–	
Pitavastatin	1	0.44 (0.23, 0.65)		1	0.44 (0.15, 0.74)		0	–	
Not indicated	14	0.73 (0.63, 0.83)		3	0.55 (0.31, 0.79)		5	0.72 (0.51, 0.94)	
Study regions			.64			.25			.92
America	9	0.74 (0.60, 0.88)		5	0.99 (0.42, 1.56)		0	–	
Europe	5	0.67 (0.40, 0.95)		0	–		3	0.78 (0.42, 1.13)	
Asia	10	0.80 (0.70, 0.90)		2	0.60 (0.24, 0.96)		4	0.80 (0.63, 0.97)	
Study quality			.18			.26			.05
>=8	15	0.77 (0.73, 0.81)		5	0.83 (0.63, 1.04)		4	0.92 (0.78, 1.07)	
<8	9	0.65 (0.49, 0.82)		2	0.61 (0.27, 0.94)		1	0.55 (0.31, 0.99)	
Cancer types			.67			.96			.73
Digestive system	8	0.80 (0.66, 0.95)		0	–		2	0.75 (0.38, 1.13)	
Respiratory system	5	0.76 (0.72, 0.79)		1	0.77 (0.73, 0.81)		2	0.83 (0.57, 1.08)	
Reproductive system	8	0.67 (0.49, 0.85)		6	0.77 (0.56, 0.97)		1	1.10 (0.50, 1.70)	
Others	3	0.81 (0.62, 0.99)		0	–		2	0.70 (0.28, 1.12)	
Statin medication			.52			.30			.22
Using alone	14	0.77 (0.67, 0.87)		5	0.81 (0.63, 1.00)		1	0.91 (0.72, 1.11)	
Combine with other methods	11	0.71 (0.56, 0.86)		2	0.60 (0.24, 0.96)		6	0.74 (0.55, 0.93)	

Digestive system: hepatocellular carcinoma, gastric cancer, and pancreatic cancer.

Respiratory system: lung cancer.

Reproductive system: ovarian cancer and prostate cancer.

Other: glioma.

## Discussion

In this systematic review and meta-analysis, statin treatment was associated with a decreased risk of overall mortality, cancer-specific survival and progression-free mortality for advanced-stage cancer patients. The associations were not attenuated or reinforced by study regions, cancer types and other medical care, except for the statin types. Concomitant use of other anticancer medications did not result in confounding effects. The results of this meta-analysis help to clarify the effects of statins on cancer survival in advanced-stage cancer patients and promote more eligible randomized trials with large sample sizes to be performed in the future.

The underlying mechanisms responsible for the reduced mortality by statins for advanced-stage cancer were attributable mainly to growth suppression, apoptosis induction, and antimetastatic effects. First, previous *in vitro* studies have demonstrated that statins can halt cancer cell proliferation by inducing G0/G1 or G2/M arrest. The involved pathways include reduction of CDK4/6 and cyclin D1 (54), blocking the CDK2/cyclin E-mediated G1/S transition (55), preventing the DNA-binding activity of NF-ĸB (56), and inhibiting DNA methyltransferases (57). Second, the induction of apoptosis was ascribed to decreased protein levels of anti-apoptotic proteins such as Bcl-2 and Bcl-xL (58, 59) and the activation of pro-apoptotic molecules such as Bax, Bad, and Caspases 3, 8, and 9 (58–61). In particular, our latest studies further explored the underlying mechanism of inducing apoptosis by statins, it was found that the depletion of GGPP rather than FPP blocked macropinocytosis, which serves as an important route for tumor nutrient uptake. Defects in macropinocytosis by statins result in protein and amino acid starvation, which further induces apoptosis (17). Finally, because metastases at distant sites rather than the primary tumors cause the majority of patients’ death (62), inhibition of metastasis by statins accounts important for the inverse association that we observed between statin use and the mortality of advanced-stage cancer. It was reported that the depletion of GGPP by statins blocks posttranslational modification of multiple small GTPase proteins to localize to the membrane. These small GTPase proteins such as RhoA, Ras and Rac are involved in cell migration and tumor invasiveness (17, 18).

We included 11 studies using other medications combined with statins for therapy, so there is the possibility of confounding effects from other treatments. However, we were able to control the suspected factors that may co-occur with the use of statins by performing subgroup analysis. Comparing the groups using statins alone with the groups using combination therapy, although the hazard ratios for using statins alone were slightly higher than those of the combination groups in the two types of survival analyses, there was no significant difference, indicating a lack of confounding effect by using other medications together with statins.

In the subgroup analysis, we were able to evaluate several potential factors that may affect the inverse association between statin therapy and mortality in advanced-stage cancer. RCT studies are commonly supposed to provide more robust evidence for meta-analysis, but in the subgroup analysis of study design, observational studies showed lower HR. Possible reasons include that these trials were designed to estimate the improvement in the effectiveness of other first-line drugs, not the effectiveness of statins alone, and more RCTs are needed to validate the robustness of our findings. In addition, accumulating data suggested that lipophilic statins provide a stronger protective effect than hydrophilic statins (63–65). However, the hydrophilic group displayed a lower hazard ratio in this study, probably because that most of the observational studies did not clarify statin types in their studies, which limited the number of included studies in this subgroup analysis. For the same reason, the significant differences we observed between each generic statin were not powerful enough to provide clinical implications, but these might be attributed to variations in pharmacokinetic properties, dosage and treatment duration, genetic factors, concomitant other medications, or patient population diversity. In terms of other factors, such as study regions, study quality, and cancer types, no significant differences were demonstrated except for the groups that included limited numbers of studies. Therefore, the subgroup analyses indicated that most of these factors did not attenuate or reinforce the association between statin treatment and the outcomes of advanced-stage cancer.

Although the protective effects of statins associated with survival rates in advanced cancer patients are lower than the approved immunotherapy medications such as PD-1 or PD-L1 inhibitors (avelumab, atezolizumab, durvalumab, nivolumab, and pembrolizumab) (66–69), which display hazard ratios of 0.57 for PD-L1 positive patients when compared with the conventional chemotherapy group (66), Statins have significant advantages as antitumour drugs. First, current lipid guidelines recommend the use of statins to reduce LDL cholesterol, and people with a history of cardiovascular disease or high LDL cholesterol are more likely to receive statins without extensive clinical safety evaluation. Second, statins are much less expensive than immunotherapy drugs. On a global scale, their cost-effectiveness could have a dramatic impact on health. In addition, our study supports the conduct of clinical trials to test the synergistic effect of statins with other approved anti-tumour drugs. In addition, some studies have reported a high risk of cardiovascular disease in cancer patients due to the cardiotoxicity of cancer therapy (70, 71). However, more studies are needed to evaluate whether statins can reduce these complications in cancer patients.

Identification of the optimal dose of statins to achieve more effectiveness in reducing cancer mortality remains a key challenge. For most of the studies included in this meta-analysis, statins were administered at a dose of 10-40 mg per day, which was the moderate-intensity statin therapy dose recommended by the American College of Cardiology/American Heart Association (ACC/AHA) for the management of blood cholesterol (72). High-intensity statin therapy has rarely been investigated for advanced-stage cancer patients, probably because a higher dose of statins (80 mg simvastatin per day) increases the risk of myopathy in myocardial infarction patients (73). In addition, a study from Denmark revealed that the cancer related mortality for overall stages did not appear to decrease as the statin dose increased (7). However, basic studies indicated that a higher dose of statins killed cancer cells more efficiently (17, 18). Therefore, more trials are needed to clarify whether the effects of reducing mortality by statins are dose dependent in advanced stage cancer patients.

Several limitations of our study need to be considered. First, there was significant heterogeneity in the magnitude of association across studies, which could be due to systemic differences in the study design, study location, characteristics of study populations, statin types, stain half-life, metabolic site, and hydrophilicity and cancer types. Nevertheless, in the sensitivity analysis excluding each study, our overall pooled effect estimates remained similar, adding to the internal validity of the conclusions. Second, there was a lack of evidence on longitudinal associations between statin therapy and survival rate in advanced-stage cancer, probably because of the considerably short survival time for advanced cancer patients. Third, a large population of randomized clinical trials with available individual participant data are required for reliable assessments of the association between different statins and survival rates for advanced cancer patients.

In conclusion, statin therapy produces significant benefits in overall survival and cancer-specific survival, irrespective of study design, study regions, cancer types and other medical care. There is low-level evidence about the efficacy of statins on progression-free survival in advanced-stage cancer. The concomitant use of statins drugs as a cancer treatment may be considered in future clinical trials.

## Data availability statement

The original contributions presented in the study are included in the article/**Supplementary Material**. Further inquiries can be directed to the corresponding authors.

## Author contributions

All authors were involved in the study design, data interpretation, and wrote the paper. ZJ and YL were responsible for searching the scientific literature, figure generation, data collection, data analysis and data screening. QZ was responsible for drafting the manuscript. ZZ and PD supervised this project.

## References

[1] Endo A . A historical perspective on the discovery of statins. Proc Jpn Acad Ser B Phys Biol Sci (2010) 86:484–93. doi: 10.2183/pjab.86.484 PMC3108295 20467214

[2] Sacks FM Pfeffer MA Moye LA Rouleau JL Rutherford JD Cole TG . The effect of pravastatin on coronary events after myocardial infarction in patients with average cholesterol levels. Cholesterol and Recurrent Events Trial investigators. N Engl J Med (1996) 335:1001–9. doi: 10.1056/NEJM199610033351401 8801446

[3] Shepherd J Blauw GJ Murphy MB Bollen EL Buckley BM Cobbe SM . Pravastatin in elderly individuals at risk of vascular disease (PROSPER): a randomised controlled trial. Lancet (2002) 360:1623–30. doi: 10.1016/S0140-6736(02)11600-X 12457784

[4] Bardou M Barkun A Martel M . Effect of statin therapy on colorectal cancer. Gut (2010) 59:1572–85. doi: 10.1136/gut.2009.190900 20660702

[5] Beckwitt CH Brufsky A Oltvai ZN Wells A . Statin drugs to reduce breast cancer recurrence and mortality. Breast Cancer Res (2018) 20:144. doi: 10.1186/s13058-018-1066-z 30458856 PMC6247616

[6] Boegemann M Schlack K Fischer AK Gerß J Steinestel J Semjonow A . Influence of statins on survival outcome in patients with metastatic castration resistant prostate cancer treated with abiraterone acetate. PloS One (2016) 11:e0161959. doi: 10.1371/journal.pone.0161959 27583544 PMC5008748

[7] Nielsen SF Nordestgaard BG Bojesen SE . Statin use and reduced cancer-related mortality. N Engl J Med (2012) 367:1792–802. doi: 10.1056/NEJMoa1201735 23134381

[8] Poynter JN Gruber SB Higgins PD Almog R Bonner JD Rennert HS . Statins and the risk of colorectal cancer. N Engl J Med (2005) 352:2184–92. doi: 10.1056/NEJMoa043792 15917383

[9] Larsson O . HMG-CoA reductase inhibitors: Role in normal and Malignant cells. Crit Rev Oncol Hemat. (1996) 22:197–212. doi: 10.1016/1040-8428(96)00193-X 8793275

[10] Sun Y Sukumaran P Varma A Derry S Sahmoun AE Singh BB . Cholesterol-induced activation of TRPM7 regulates cell proliferation, migration, and viability of human prostate cells. Biochim Biophys Acta (2014) 1843:1839–50. doi: 10.1016/j.bbamcr.2014.04.019 PMC4096426 24769209

[11] dos Santos CR Domingues G Matias I Matos J Fonseca I de Almeida JM . LDL-cholesterol signaling induces breast cancer proliferation and invasion. Lipids Health Dis (2014) 13:16. doi: 10.1186/1476-511X-13-16 24428917 PMC3896822

[12] Bifulco M . Role of the isoprenoid pathway in ras transforming activity, cytoskeleton organization, cell proliferation and apoptosis. Life Sci (2005) 77:1740–9. doi: 10.1016/j.lfs.2005.05.017 15955538

[13] Zhuang LY Kim J Adam RM Solomon KR Freeman MR . Cholesterol targeting alters lipid raft composition and cell survival in prostate cancer cells and xenografts. J Clin Invest. (2005) 115:959–68. doi: 10.1172/JCI200519935 PMC1064980 15776112

[14] Zhuang LY Lin JQ Lu ML Solomon KR Freeman MR . Cholesterol-rich lipid rafts mediate Akt-regulated survival in prostate cancer cells. Cancer Res (2002) 62:2227–31. 11956073

[15] Ingallina E Sorrentino G Bertolio R Lisek K Zannini A Azzolin L . Mechanical cues control mutant p53 stability through a mevalonate-RhoA axis. Nat Cell Biol (2018) 20:28–+. doi: 10.1038/s41556-017-0009-8 PMC6179142 29255172

[16] Sorrentino G Ruggeri N Specchia V Cordenonsi M Mano M Dupont S . Metabolic control of YAP and TAZ by the mevalonate pathway. Nat Cell Biol (2014) 16:357–66. doi: 10.1038/ncb2936 24658687

[17] Jiao Z Cai H Long Y Sirka OK Padmanaban V Ewald AJ . Statin-induced GGPP depletion blocks macropinocytosis and starves cells with oncogenic defects. Proc Natl Acad Sci U S A. (2020) 117:4158–68. doi: 10.1073/pnas.1917938117 PMC7049144 32051246

[18] Freed-Pastor WA Mizuno H Zhao X Langerød A Moon SH Rodriguez-Barrueco R . Mutant p53 disrupts mammary tissue architecture via the mevalonate pathway. Cell (2012) 148:244–58. doi: 10.1016/j.cell.2011.12.017 PMC3511889 22265415

[19] Sanfilippo KM Keller J Gage BF Luo S Wang TF Moskowitz G . Statins are associated with reduced mortality in multiple myeloma. J Clin Oncol (2016) 34:4008–14. doi: 10.1200/JCO.2016.68.3482 PMC5477827 27646948

[20] Iarrobino NA Gill B Bernard ME Mishra MV Champ CE . Targeting tumor metabolism with statins during treatment for advanced-stage pancreatic cancer. Am J Clin Oncol-Canc. (2018) 41:1125–31. doi: 10.1097/COC.0000000000000433 PMC6123314 29509593

[21] Platz EA Leitzmann MF Visvanathan K Rimm EB Stampfer MJ Willett WC . Statin drugs and risk of advanced prostate cancer. J Natl Cancer Inst (2006) 98:1819–25. doi: 10.1093/jnci/djj499 17179483

[22] Jouve JL Lecomte T Bouche O Barbier E Khemissa Akouz F Riachi G . Pravastatin combination with sorafenib does not improve survival in advanced hepatocellular carcinoma. J Hepatol (2019) 71:516–22. doi: 10.1016/j.jhep.2019.04.021 31125576

[23] Bujanda L Rodriguez-Gonzalez A Sarasqueta C Eizaguirre E Hijona E Marín JJ . Effect of pravastatin on the survival of patients with advanced gastric cancer. Oncotarget (2016) 7:4379–84. doi: 10.18632/oncotarget.6777 PMC4826212 26735890

[24] Chen H-Y Wang Q Xu Q-H Yan L Gao XF Lu YH . Statin as a combined therapy for advanced-stage ovarian cancer: A propensity score matched analysis. BioMed Res Int (2016) 2016:9125238. doi: 10.1155/2016/9125238 27975064 PMC5128698

[25] Kawata S Yamasaki E Nagase T Inui Y Ito N Matsuda Y . Effect of pravastatin on survival in patients with advanced hepatocellular carcinoma. A randomized controlled trial. Br J Cancer. (2001) 84:886–91. doi: 10.1054/bjoc.2000.1716 PMC2363838 11286466

[26] Yang H Pang L Hu X Wang W Xu B Zhang X . The effect of statins on advanced prostate cancer patients with androgen deprivation therapy or abiraterone/enzalutamide: A systematic review and meta-analysis. J Clin Pharm Ther (2020) 45:488–95. doi: 10.1111/jcpt.13092 31951037

[27] Stroup DF Berlin JA Morton SC Olkin I Williamson GD Rennie D . Meta-analysis of observational studies in epidemiology: a proposal for reporting. Meta-analysis Of Observational Studies in Epidemiology (MOOSE) group. JAMA (2000) 283:2008–12. doi: 10.1001/jama.283.15.2008 10789670

[28] Chou R Dana T Blazina I Daeges M Jeanne TL . Statins for prevention of cardiovascular disease in adults: evidence report and systematic review for the US preventive services task force. JAMA (2016) 316:2008–24. doi: 10.1001/jama.2015.15629 27838722

[29] Han JY Lee SH Yoo NJ Hyung LS Moon YJ Yun T . A randomized phase II study of gefitinib plus simvastatin versus gefitinib alone in previously treated patients with advanced non-small cell lung cancer. Clin Cancer Res (2011) 17:1553–60. doi: 10.1158/1078-0432.CCR-10-2525 21411446

[30] Kim ST Kang JH Lee J Park SH Park JO Park YS . Simvastatin plus capecitabine-cisplatin versus placebo plus capecitabine-cisplatin in patients with previously untreated advanced gastric cancer: a double-blind randomised phase 3 study. Eur J Cancer. (2014) 50:2822–30. doi: 10.1016/j.ejca.2014.08.005 25218337

[31] Lee Y Lee KH Lee GK Lee SH Lim KY Joo J . Randomized phase II study of afatinib plus simvastatin versus afatinib alone in previously treated patients with advanced nonadenocarcinomatous non-small cell lung cancer. Cancer Res Treat (2017) 49:1001–11. doi: 10.4143/crt.2016.546 PMC5654166 28111428

[32] Alarfi H Youssef LA Salamoon M . A prospective, randomized, placebo-controlled study of a combination of simvastatin and chemotherapy in metastatic breast cancer. J Oncol (2020) 2020:4174395. doi: 10.1155/2020/4174395 32849871 PMC7436279

[33] Wu SY Fang SC Shih HJ Wen YC Shao YHJ . Mortality associated with statins in men with advanced prostate cancer treated with androgen deprivation therapy. Eur J Cancer. (2019) 112:109–17. doi: 10.1016/j.ejca.2018.11.032 30827745

[34] Gordon JA Buonerba C Pond G Crona D Gillessen S Lucarelli G . Statin use and survival in patients with metastatic castration-resistant prostate cancer treated with abiraterone or enzalutamide after docetaxel failure: the international retrospective observational STABEN study. Oncotarget (2018) 9:19861–73. doi: 10.18632/oncotarget.24888 PMC5929432 29731989

[35] Di Lorenzo G Sonpavde G Pond G Lucarelli G Rossetti S Facchini G . Statin use and survival in patients with metastatic castration-resistant prostate cancer treated with abiraterone acetate. Eur Urol Focus. (2018) 4:874–9. doi: 10.1016/j.euf.2017.03.015 28753882

[36] Jung J Lee C Lee C Kwon T You D Jeong IG . Effects of statin use on the response duration to androgen deprivation therapy in metastatic prostate cancer. Korean J Urol. (2015) 56:630–6. doi: 10.4111/kju.2015.56.9.630 PMC4565897 26366275

[37] Lopez DS Huang D Tsilidis KK Canfield S Khera M Baillargeon JG . The role of testosterone replacement therapy and statin use, and their combination, in prostate cancer. Cancer Causes Control. (2021) 32:965–76. doi: 10.1007/s10552-021-01450-0 PMC8316375 34041642

[38] Khan S Chang S-H Hicks V Wang M Grubb RL Drake BF . Improved survival with post-diagnostic metformin and statin use in a racially diverse cohort of US Veterans with advanced prostate cancer. Prostate Cancer prostatic diseases. (2021) 25(4):707–12. doi: 10.1038/s41391-021-00475-5 34811499

[39] Kumar A Riviere P Luterstein E Nalawade V Vitzthum L Sarkar RR . Associations among statins, preventive care, and prostate cancer mortality. Prostate Cancer prostatic diseases. (2020) 23:475–85. doi: 10.1038/s41391-020-0207-5 32029930

[40] Nakai Y Isayama H Sasaki T Mizuno S Sasahira N Kogure H . Clinical outcomes of chemotherapy for diabetic and nondiabetic patients with pancreatic cancer better prognosis with statin use in diabetic patients. Pancreas (2013) 42:202–8. doi: 10.1097/MPA.0b013e31825de678 23000889

[41] Jeon CY Pandol SJ Wu B Cook-Wiens G Gottlieb RA Merz CN . The association of statin use after cancer diagnosis with survival in pancreatic cancer patients: a SEER-medicare analysis. PloS One (2015) 10:e0121783. doi: 10.1371/journal.pone.0121783 25830309 PMC4382214

[42] Moon DC Lee HS Lee YI Chung MJ Park JY Park SW . Concomitant statin use has a favorable effect on gemcitabine-erlotinib combination chemotherapy for advanced pancreatic cancer. Yonsei Med J (2016) 57:1124–30. doi: 10.3349/ymj.2016.57.5.1124 PMC4960377 27401642

[43] Hamada T Khalaf N Yuan C Morales-Oyarvide V Babic A Nowak JA . Prediagnosis use of statins associates with increased survival times of patients with pancreatic cancer. Clin Gastroenterol H. (2018) 16:1300–+. doi: 10.1016/j.cgh.2018.02.022 PMC6056316 29474971

[44] Lin JJ Ezer N Sigel K Mhango G Wisnivesky JP . The effect of statins on survival in patients with stage IV lung cancer. Lung Cancer. (2016) 99:137–42. doi: 10.1016/j.lungcan.2016.07.006 PMC5003323 27565929

[45] Lam VK Bentzen SM Mohindra P Nichols EM Bhooshan N Vyfhuis M . Obesity is associated with long-term improved survival in definitively treated locally advanced non-small cell lung cancer (NSCLC). Lung Cancer. (2017) 104:52–7. doi: 10.1016/j.lungcan.2016.11.017 28213000

[46] Takada K Shimokawa M Takamori S Shimamatsu S Hirai F Tagawa T . A propensity score-matched analysis of the impact of statin therapy on the outcomes of patients with non-small-cell lung cancer receiving anti-PD-1 monotherapy: a multicenter retrospective study. BMC Cancer. (2022) 22:503. doi: 10.1186/s12885-022-09385-8 35524214 PMC9074359

[47] Shao JYH Lee FP Chang CL Wu SY . Statin-based palliative therapy for hepatocellular carcinoma. Medicine (2015) 94(42):e1801. doi: 10.1097/MD.0000000000001801 26496314 PMC4620768

[48] Gonzalez R Gockley AA Melamed A Sugrue R Clark RM Del Carmen MG . Multivariable analysis of association of beta-blocker use and survival in advanced ovarian cancer. Gynecologic Oncol (2020) 157:700–5. doi: 10.1016/j.ygyno.2020.03.012 32222327

[49] Elmore RG Ioffe Y Scoles DR Karlan BY Li AJ . Impact of statin therapy on survival in epithelial ovarian cancer. Gynecol Oncol (2008) 111:102–5. doi: 10.1016/j.ygyno.2008.06.007 20698078

[50] Dighe SG Yan L Mukherjee S McGillicuddy CS Hulme KL Hochwald SN . Clinical and lifestyle-related prognostic indicators among esophageal adenocarcinoma patients receiving treatment at a comprehensive cancer center. Cancers (2021) 13(18):4653. doi: 10.3390/cancers13184653 34572881 PMC8465866

[51] Seliger C Schaertl J Gerken M Luber C Proescholdt M Riemenschneider MJ . Use of statins or NSAIDs and survival of patients with high-grade glioma. PloS One (2018) 13(12):e0207858. doi: 10.1371/journal.pone.0207858 30507932 PMC6277074

[52] Santoni M Molina-Cerrillo J Myint ZW Massari F Buchler T Buti S . Concomitant use of statins, metformin, or proton pump inhibitors in patients with advanced renal cell carcinoma treated with first-line combination therapies. Target Oncol (2022) 17:571–81. doi: 10.1007/s11523-022-00907-9 35947324

[53] Min Y Liu Z Wei Z Li R Jin J Zhang Y . Association between statin use and survival in cancer patients with brain metastasis: retrospective analysis from the chinese population. Pharm (Basel). (2022) 15(12):1474. doi: 10.3390/ph15121474 PMC9781124 36558925

[54] Wang G Cao R Wang Y Qian G Dan HC Jiang W . Simvastatin induces cell cycle arrest and inhibits proliferation of bladder cancer cells via PPARgamma signalling pathway. Sci Rep (2016) 6:35783. doi: 10.1038/srep35783 27779188 PMC5078845

[55] Sivaprasad U Abbas T Dutta A . Differential efficacy of 3-hydroxy-3-methylglutaryl CoA reductase inhibitors on the cell cycle of prostate cancer cells. Mol Cancer Ther (2006) 5:2310–6. doi: 10.1158/1535-7163.MCT-06-0175 16985065

[56] Gazzerro P Proto MC Gangemi G Malfitano AM Ciaglia E Pisanti S . Pharmacological actions of statins: a critical appraisal in the management of cancer. Pharmacol Rev (2012) 64:102–46. doi: 10.1124/pr.111.004994 22106090

[57] Karlic H Thaler R Gerner C Grunt T Proestling K Haider F . Inhibition of the mevalonate pathway affects epigenetic regulation in cancer cells. Cancer Genet (2015) 208:241–52. doi: 10.1016/j.cancergen.2015.03.008 PMC4503872 25978957

[58] Goc A Kochuparambil ST Al-Husein B Al-Azayzih A Mohammad S SOmanath PR . Simultaneous modulation of the intrinsic and extrinsic pathways by simvastatin in mediating prostate cancer cell apoptosis. BMC Cancer. (2012) 12:409. doi: 10.1186/1471-2407-12-409 22974127 PMC3522038

[59] Spampanato C De Maria S Sarnataro M Giordano E Zanfardino M Baiano S . Simvastatin inhibits cancer cell growth by inducing apoptosis correlated to activation of Bax and down-regulation of BCL-2 gene expression. Int J Oncol (2012) 40:935–41. doi: 10.3892/ijo.2011.1273 PMC3584570 22134829

[60] Buranrat B Suwannaloet W Naowaboot J . Simvastatin potentiates doxorubicin activity against MCF-7 breast cancer cells. Oncol Lett (2017) 14:6243–50. doi: 10.3892/ol.2017.6783 PMC5661424 29113274

[61] Kotamraju S Williams CL Kalyanaraman B . Statin-induced breast cancer cell death: role of inducible nitric oxide and arginase-dependent pathways. Cancer Res (2007) 67:7386–94. doi: 10.1158/0008-5472.CAN-07-0993 17671209

[62] Weigelt B Peterse JL van 't Veer LJ . Breast cancer metastasis: markers and models. Nat Rev Cancer. (2005) 5:591–602. doi: 10.1038/nrc1670 16056258

[63] Rutledge BP Desai P Liu S Luo J Nassir R Lihong Q . The association between statins and colorectal cancer stage in the Women's Health Initiative. Mol Clin Oncol (2019) 11:252–8. doi: 10.3892/mco.2019.1895 PMC6688425 31423310

[64] Manthravadi S Shrestha A Madhusudhana S . Impact of statin use on cancer recurrence and mortality in breast cancer: A systematic review and meta-analysis. Int J Cancer. (2016) 139:1281–8. doi: 10.1002/ijc.30185 27176735

[65] Liu B Yi Z Guan X Zeng YX Ma F . The relationship between statins and breast cancer prognosis varies by statin type and exposure time: a meta-analysis. Breast Cancer Res Treat (2017) 164:1–11. doi: 10.1007/s10549-017-4246-0 28432513

[66] Bellmunt J de Wit R Vaughn DJ Fradet Y Lee JL Fong L . Pembrolizumab as second-line therapy for advanced urothelial carcinoma. N Engl J Med (2017) 376:1015–26. doi: 10.1056/NEJMoa1613683 PMC5635424 28212060

[67] Borghaei H Paz-Ares L Horn L Spigel DR Steins M Ready NE . Nivolumab versus docetaxel in advanced nonsquamous non-small-cell lung cancer. N Engl J Med (2015) 373:1627–39. doi: 10.1056/NEJMoa1507643 PMC5705936 26412456

[68] Reck M Rodriguez-Abreu D Robinson AG Hui R Csőszi T Fülöp A . Pembrolizumab versus chemotherapy for PD-L1-positive non-small-cell lung cancer. N Engl J Med (2016) 375:1823–33. doi: 10.1056/NEJMoa1606774 27718847

[69] Shen X Zhao B . Efficacy of PD-1 or PD-L1 inhibitors and PD-L1 expression status in cancer: meta-analysis. BMJ (2018) 362:k3529. doi: 10.1136/bmj.k3529 30201790 PMC6129950

[70] Floyd JD Nguyen DT Lobins RL Bashir Q Doll DC Perry MC . Cardiotoxicity of cancer therapy. J Clin Oncol (2005) 23:7685–96. doi: 10.1200/JCO.2005.08.789 16234530

[71] Khakoo AY Liu PP Force T Lopez-Berestein G Jones LW Schneider J . Cardiotoxicity due to cancer therapy. Tex Heart Inst J (2011) 38:253–6. PMC3113124 21720463

[72] Grundy SM Stone NJ Bailey AL Beam C Birtcher KK Blumenthal RS . 2018 AHA/ACC/AACVPR/AAPA/ABC/ACPM/ADA/AGS/APhA/ASPC/NLA/PCNA guideline on the management of blood cholesterol: executive summary: A report of the american college of cardiology/american heart association task force on clinical practice guidelines. Circulation (2019) 139:e1046–81. doi: 10.1161/CIR.0000000000000624 30565953

[73] Study of the Effectiveness of Additional Reductions in C, Homocysteine Collaborative G Armitage J Bowman L Wallendszus K Bulbulia R Rahimi K . Intensive lowering of LDL cholesterol with 80 mg versus 20 mg simvastatin daily in 12,064 survivors of myocardial infarction: a double-blind randomised trial. Lancet (2010) 376:1658–69. doi: 10.1016/S0140-6736(10)60310-8 PMC2988223 21067805

