# Supplementary figures and images for: The effects of statins in patients with advanced-stage cancers - a systematic review and meta-analysis
Source: Front Oncol. 2023 Aug 18;13:1234713. doi: 10.3389/fonc.2023.1234713 (PMC10473877; doi:10.3389/fonc.2023.1234713)

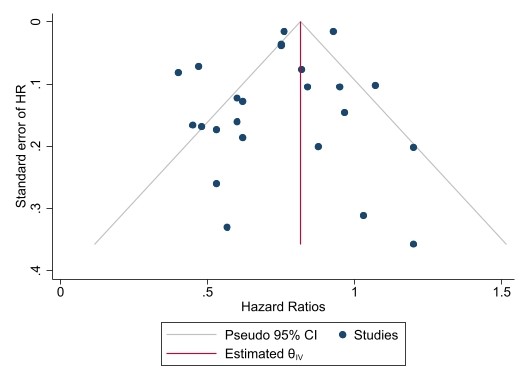

Supplement: Supplementary file 1 [file Image_1.jpeg]

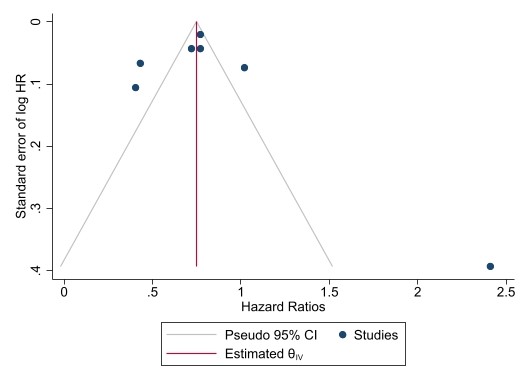

Supplement: Supplementary file 2 [file Image_2.jpeg]

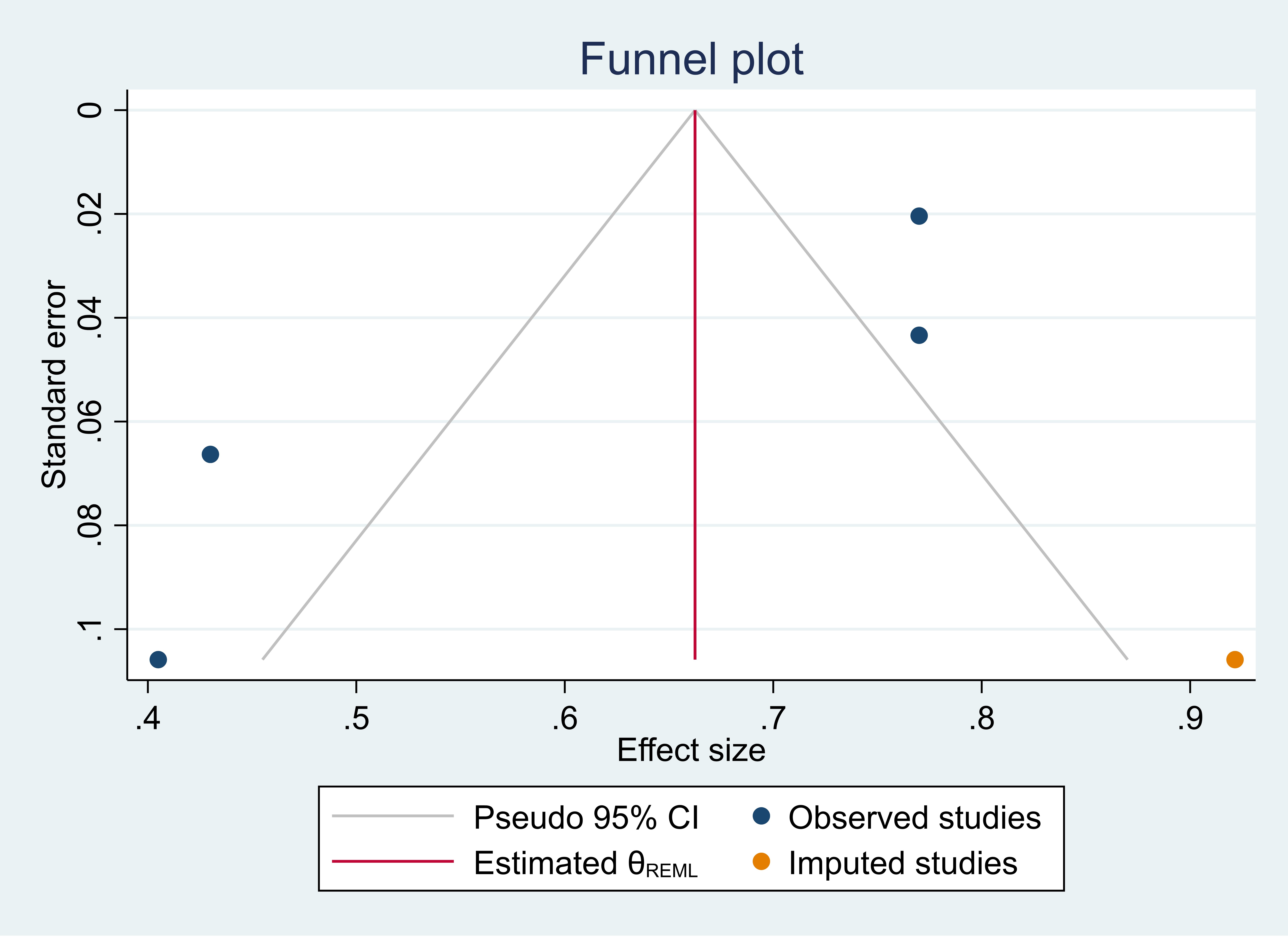

Supplement: Supplementary file 3 [file Image_3.jpeg]

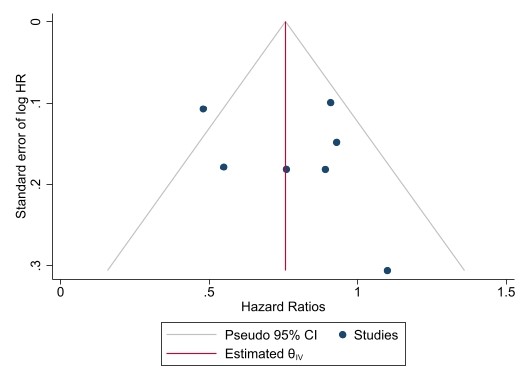

Supplement: Supplementary file 4 [file Image_4.jpeg]
