# Supplementary material for: The effects of statins in patients with advanced-stage cancers - a systematic review and meta-analysis
Source: Front Oncol. 2023 Aug 18;13:1234713. doi: 10.3389/fonc.2023.1234713 (PMC10473877; doi:10.3389/fonc.2023.1234713)
Supplement: Supplementary file 5 [file DataSheet_1.docx]

**Contents**

Searching Strategies

Quality Assessment and Supplementary Analyses

Textbox 1. Inclusion and exclusion criteria

Table S1. New Castle-Ottawa Scoring for non-randomized studies.

Table S2. Quality rating of randomized controlled trails.

Table S3. Sensitive analysis of the association between statin usage and overall survival in the advanced-stage cancer patients.

Table S4. Sensitive analysis of the association between statin usage and cancer-specific survival in the advanced-stage cancer patients.

Table S5. Sensitive analysis of the association between statin usage and progression-free survival in the advanced-stage cancer patients.

Figure S1. Funnel plot for the effect of statin use on overall survival in patients with advanced-stage cancers.

Figure S2. Funnel plot for the effect of statin use on cancer-specific survival in patients with advanced-stage cancers.

Figure S3. Funnel plot for the effect of statin use on cancer-specific survival in advanced-stage cancers patients with imputed the omitted studies.

Figure S4. Funnel plot for the effect of statin use on free-progression survival in patients with advanced-stage cancers.

**Searching strategies:**

**Medline**:

|  |  | n |
| --- | --- | --- |
| #1 | (((((((((“advance”[All Fields] OR “advanced”[All Fields]) OR “advancement”[All Fields]) OR “advancements”[All Fields]) OR “advances”[All Fields]) OR “advancing”[All Fields]) OR “high-risk”[All Fields]) OR “high-grade”[All Fields]) OR (((((“stage”[All Fields] OR “staged”[All Fields]) OR “stages”[All Fields]) OR “staging”[All Fields]) OR “stagings”[All Fields]) AND “III”[All Fields])) OR (((((“stage”[All Fields] OR “staged”[All Fields]) OR “stages”[All Fields]) OR “staging”[All Fields]) OR “stagings”[All Fields]) AND “IV”[All Fields])) AND (((“Neoplasms”[MeSH Terms] OR “Neoplasms”[Text Word]) ) OR “Cancer”[Text Word]) | 547,067 |
| #2 | ("Hydroxymethylglutaryl-CoA Reductase Inhibitors" [Pharmacological Action] OR "Hydroxymethylglutaryl-CoA Reductase Inhibitors" [Mesh] OR "Hydroxymethylglutaryl-CoA Reductase Inhibitors" [tw] OR "Fluvastatin"[Mesh] OR "Fluvastatin"[tw] OR "Rosuvastatin Calcium"[Mesh] OR "Rosuvastatin Calcium" [tw] OR "Atorvastatin"[Mesh] OR "Atorvastatin"[tw] OR "Lovastatin"[Mesh] OR "Lovastatin"[tw] OR "Simvastatin"[Mesh] OR "Simvastatin"[tw] OR "Pravastatin"[Mesh] OR "Pravastatin"[tw] OR "Cerivastatin"[tw] OR "statin" [tw]) | 60,992 |
| #3 | #1 AND #2 | 607 |
| #4 | ("Animals"[MeSH] NOT "Humans"[MeSH]) | 5.094.539 |
| #5 | #3 NOT #4 | 611 |

EMBASE

| #1 | 'hydroxymethylglutaryl coenzyme a' | 141,178 |
| --- | --- | --- |
| #2 | 'fluindostatin':ab,ti,kw OR 'rosuvastatin':ab,ti,kw OR 'atorvastatin':ab,ti,kw OR 'mevinolin':ab,ti,kw OR 'simvastatin':ab,ti,kw OR 'pravastatin':ab,ti,kw OR 'cerivastatin':ab,ti,kw | 38,920 |
| #3 | #1 OR #2 | 169,371 |
| #4 | 'neoplasm':ab,ti,kw OR 'malignant neoplasm':ab,ti,kw OR 'cancer':ab,ti,kw | 3,163,156 |
| #5 | advanc*:ab,ti,kw OR 'high risk':ab,ti,kw OR 'high grade':ab,ti,kw OR 'stag* iii':ab,ti,kw OR 'stag* iv':ab,ti,kw | 2,113,117 |
| #6 | #4 AND #5 | 565,836 |
| #7 | #3 AND #6 | 1,767 |
| #8 | 'animal'/exp NOT 'human'/exp | 6,155,364 |
| #9 | #7 NOT #8 | 1,885 |

**Web of Science**

|  |  | n |
| --- | --- | --- |
| #1 | (Hydroxymethylglutaryl-CoA Reductase Inhibitors OR Fluvastatin OR Rosuvastatin Calcium OR Atorvastatin OR Lovastatin OR Simvastatin OR Pravastatin OR Cerivastatin OR statin) | 101,031 |
| #2 | (Advance* OR ‘High risk’ OR ‘High grade’ OR “Stag* III” OR “Stag* IV”) AND ('neoplasm' OR 'malignant neoplasm' OR ‘Cancer’) | 1,004,051 |
| #3 | #1 AND #2 | 1,561 |

**Quality Assessment**

Quality assessment in observational research is controversial, with no clear consensus on rating methods or their appropriate use in the analysis. We adopted the Newcastle-Ottawa Scale to evaluate non-randomized studies ([http://www.ohri.ca/programs/clinical epidemiology/oxf ord.asp](http://www.ohri.ca/programs/clinical%20epidemiology/oxf%20ord.asp)). This scale assigns up to 4 points for a low risk of bias in the selection of patients and comparators, 2 possible points for comparability, and 3 points for a low risk of bias in the determination of exposure. The scale evaluates the quality of observational studies, allocating a maximum of 9 stars for higher quality and lower risk of bias.

We adopted the US Preventive Services Task Force Procedure Manual to evaluate randomized clinical trials.^1^ The scale rate the quality of RCT studies as good, fair, or poor. The quality ratings and definitions describe as follows:

Good: Evidence consists of studies without important limitations in study design, quality, or precision; consistent findings across studies; and highly applicable to U.S. primary care settings. More evidence is unlikely to change the magnitude or direction of findings.

Fair: Evidence consists of studies with some limitations in study design, quality or precision; some inconsistency across studies; and/or limited applicability to U.S. primary care settings. More evidence may change the magnitude or direction of the findings.

Poor: Evidence consists of studies with serious limitations in study design, quality, or precision; serious inconsistency across studies; and/or poor applicability to U.S. primary care settings. More evidence is likely to change the magnitude or direction of the findings.

Textbox 1. Inclusion and exclusion criteria

**Inclusion criteria**

- Population: patients with high-grade, high-stage, or advanced-stage cancer, and aged ≥18 years
- Intervention: evaluated the effects of statin regimens
- Comparison: the overall survival, cancer-specific mortality, and progression-free survival rates of patients with advanced-stage cancer who taking statins with patients with advanced-stage cancer who do not take statins.
- Outcome: 1) overall survival; 2) cancer-specific survival; and 3) progression-free survival
- Study design: observational studies and randomized controlled trials

**Exclusion criteria**

- Conference papers
- Animal studies
- Letters
- Case reports
- Commentaries and editorials
- Reviews
- Studies that did not present original data
- Studies that did not report survival rates

**Table S1**. New Castle-Ottawa scoring for non-randomized studies.

| Study | Subject Selection Max 4 | Study Comparability Max 2 | Assessment of Outcomes Max 3 | Total Score |
| --- | --- | --- | --- | --- |
| Moon, 2016 | 4 | 2 | 1 | 7 |
| Bujanda, 2016 | 4 | 2 | 3 | 9 |
| Wu, 2019 | 4 | 2 | 3 | 9 |
| Chen, 2016 | 4 | 2 | 3 | 9 |
| Shao, 2015 | 4 | 1 | 2 | 7 |
| Lin, 2016 | 4 | 2 | 1 | 7 |
| Seliger, 2018 | 4 | 2 | 3 | 9 |
| Nakai, 2013 | 4 | 2 | 1 | 7 |
| Elmore, 2008 | 4 | 2 | 1 | 7 |
| Gonzalez, 2020 | 4 | 2 | 2 | 8 |
| Lam, 2017 | 3 | 2 | 3 | 8 |
| Gordon, 2018 | 3 | 2 | 1 | 6 |
| Lorenzo, 2018 | 4 | 2 | 1 | 7 |
| Boegemann, 2016 | 4 | 2 | 3 | 9 |
| Hamada, 2018 | 3 | 2 | 3 | 8 |
| Jeon, 2015 | 4 | 2 | 1 | 7 |
| Jung, 2015 | 4 | 2 | 3 | 9 |
| Khan, 2021 | 4 | 2 | 3 | 9 |
| Dighe, 2021 | 4 | 2 | 2 | 8 |
| Lopez, 2021 | 4 | 2 | 3 | 9 |
| Kumar, 2020 | 4 | 2 | 2 | 8 |
| Santoni, 2022 | 3 | 2 | 2 | 7 |
| Min, 2022 | 4 | 2 | 3 | 9 |
| Takada, 2022 | 4 | 2 | 2 | 8 |

**Table S2.** Quality rating of randomized controlled trails.

| Study name, author, year, reference | Randomization adequate? | Allocation concealment adequate? | Groups similar at baseline? | Eligibility criteria specified | Outcome assessors masked? | Care provider masked? | Patient masked? | Attrition and withdrawals reported? | Loss to follow-up differential? | Loss to follow-up high? | Analyze people in the groups in which they were randomized? | Quality rating |
| --- | --- | --- | --- | --- | --- | --- | --- | --- | --- | --- | --- | --- |
| Han, 2011 | Yes | Not clear | Yes | Yes | Not clear | Not clear | Not clear | No | No | No | Yes | Fair |
| Kim, 2014 | Yes | Not clear | Yes | Yes | Yes | Yes | Yes | No | No | No | Yes | Good |
| Lee, 2017 | Yes | Not clear | Yes | Yes | Not clear | Not clear | Not clear | Yes | No | No | Yes | Fair |

**Table S3.** Sensitive analysis of the association between statin usage and overall survival in the advanced-stage cancer patients.

| Study Omitted | Hazard Ratio (95% CI) | Percentage of change |
| --- | --- | --- |
| Elmore, 2008, observational | 0.756 (0.683, 0.828) | 1.20% |
| Han, 2011, RCT | 0.743 (0.670, 0.817) | 0.54% |
| Nakai, 2013, observational | 0.750 (0.677, 0.823) | 0.40% |
| Kim, 2014, RCT | 0.738 (0.665, 0.812) | 1.20% |
| Jeon, 2015, observational | 0.730 (0.656, 0.803) | 2.28% |
| Shao, 2015, observational | 0.730 (0.662, 0.797) | 2.28% |
| Boegemann, 2016, observational | 0.742 (0.760, 0.815) | 0.67% |
| Moon, 2016, observational | 0.755 (0.682, 0.828) | 1.07% |
| Bujanda, 2016, observational | 0.750 (0.677, 0.823) | 0.40% |
| Chen, 2016, observational | 0.748 (0.676, 0.821) | 0.13% |
| Lin, 2016, observational | 0.746 (0.658, 0.833) | 0.13% |
| Lam, 2017, observational | 0.753 (0.679, 0.827) | 0.80% |
| Lee, 2017, RCT | 0.743 (0.670, 0.816) | 0.54% |
| Gordon, 2018, observational | 0.765 (0.694, 0.836) | 2.41% |
| Lorenzo, 2018, observational | 0.768 (0.698, 0.838) | 2.81% |
| Seliger, 2018, observational | 0.736 (0.662, 0.810) | 1.47% |
| Hamada, 2018, observational | 0.742 (0.667, 0.816) | 0.67% |
| Wu, 2019, observational | 0.746 (0.668, 0.824) | 0.13% |
| Gonzalez, 2020, observational | 0.736 (0.663, 0.808) | 1.47% |
| Khan, 2021, observational | 0.746 (0.669, 0.823) | 0.13% |
| Dighe, 2021, observational | 0.752 (0.678, 0.825) | 0.67% |
| Santoni, 2022, observational | 0.753 (0.680, 0.826) | 0.80% |
| Min, 2022, observational | 0.742 (0.666, 0.817) | 0.67% |
| Takada, 2022, observational | 0.751 (0.678, 0.825) | 0.54% |
| **Pooled Estimation** | 0.741 (0.669, 0.814) |  |

**Table S4.** Sensitive analysis of the association between statin usage and cancer-specific survival in the advanced-stage cancer patients.

| Study Omitted | Hazard Ratio (95% CI) | Percentage of change |
| --- | --- | --- |
| Jung, 2015, observational | 0.788 (0.650, 0.926) | 5.21% |
| Lin, 2016, observational | 0.765 (0.562, 0.967) | 2.14% |
| Gordon, 2018, observational | 0.792 (0.664, 0.920) | 5.74% |
| Wu, 2019, observational | 0.750 (0.575, 0.924) | 0.13% |
| Kumar, 2020, observational | 0.699 (0.579, 0.819) | 6.68% |
| Khan, 2021, observational | 0.759 (0.585, 0.934) | 1.34% |
| Lopez, 2021, observational | 0.688 (0.548, 0.828) | 8.14% |
| **Pooled Estimation** | 0.742 (0.606, 0.878) |  |

**Table S5.** Sensitive analysis of the association between statin usage and cancer-specific survival in the advanced-stage cancer patients.

| Study Omitted | Hazard Ratio (95% CI) | Percentage of change |
| --- | --- | --- |
| Han, 2011, RCT | 0.758 (0.562, 0.953) | 12.47% |
| Kim, 2014, RCT | 0.746 (0.551, 0.940) | 15.80% |
| Moon, 2016, observational | 0.807 (0.619, 0.996) | 8.92% |
| Boegemann, 2016, observational | 0.751 (0.572, 0.930) | 15.24% |
| Seliger, 2018, observational | 0.739 (0.544, 0.993) | 16.59% |
| Santoni, 2022, observational | 0.856 (0.732, 0.980) | 3.39% |
| Takada, 2022, observational | 0.788 (0.578, 0.978) | 11.06% |
| **Pooled Estimation** | 0.759 (0.652, 0.865) |  |

**Reference**

1. Chou R, Dana T, Blazina I, Daeges M, Jeanne TL. Statins for Prevention of Cardiovascular Disease in Adults: Evidence Report and Systematic Review for the US Preventive Services Task Force. *JAMA.* 2016;316(19):2008-2024.
